# Electrical Modification of Self-Assembled Polymer-Stabilized Periodic Microstructures in a Liquid Crystal Composite

**DOI:** 10.3390/polym17243342

**Published:** 2025-12-18

**Authors:** Miłosz S. Chychłowski, Marta Kajkowska, Jan Bolek, Oleksandra Gridyakina, Bartosz Bartosewicz, Bartłomiej Jankiewicz, Piotr Lesiak

**Affiliations:** 1 Faculty of Physics, Warsaw University of Technology, Koszykowa 75, 00-662 Warsaw, Poland; marta.kajkowska.dokt@pw.edu.pl (M.K.); oleksandra.gridyakina@pw.edu.pl (O.G.); piotr.lesiak@pw.edu.pl (P.L.); 2 Aerospace Faculty, National Aviation University, Liubomyra Huzara Ave. 1, 03058 Kiev, Ukraine; 3 Institute of Optoelectronics, Military University of Technology, Kaliskiego 2, 00-908 Warsaw, Poland

**Keywords:** liquid crystal, polymer-stabilized liquid crystal, self-assembled periodicity, gold nanoparticles

## Abstract

Utilization of natural processes can reduce the complexity and production cost of any device by limiting the necessary steps in the production scheme, especially when it comes to fibers with periodic changes in refractive index. One such process is the nematic–isotropic phase separation of liquid crystal-based composite confined in 1D space. In this paper, we analyze the behavior of polymer-stabilized liquid crystal-based self-assembled periodic structures in an external electric field. We performed a detailed analysis regarding the reorientation of liquid crystal molecules under two orthogonal directions of the external electric field applied to the examined sample. It was demonstrated that the period of the polymerized structure remains constant until full reorientation, as the electric field induces the formation of new periodic defects in LC orientation. Consequently, the structure’s effective birefringence changes quite drastically, and this observed change depends on the direction of the electric field vector. The obtained results seem promising when it comes to application of the proposed periodic structures as voltage or electric field sensors operating as long-period fiber gratings or fiber Bragg gratings for the visible or near-infrared spectral regions.

## 1. Introduction

Liquid crystals (LCs) have been widely used in photonic applications for several decades, mostly due to their unique electro-optical properties [1,2,3,4,5,6,7]. These materials are highly birefringent, and their optical properties can be tuned with both electric and magnetic fields [8,9,10]. Since the creation of the first LCDs in the 1960s [11], researchers have focused on modifying the properties of LCs for the purpose of improving the performance of existing LC-based devices [12], as well as for making the LC materials suitable for new applications [13]. The new properties of LCs are often achieved by adding dopants [14,15,16,17], such as nanoparticles (NPs) [3,18,19,20,21,22,23,24,25,26] or monomers [7,23,24,25,27,28,29,30,31,32,33,34,35,36], to the LC host. It was demonstrated that doping the LCs with nanoparticles can result in a reduction in threshold voltage and switching times [3,18,21], which is mostly desirable for display applications or all-fiber-based switches [37,38,39,40,41,42]. On the other hand, the polymerization of composites consisting of LCs and monomers provides higher temperature stability of the material [29,30,36] and allows for stabilization of the desired molecular arrangement of the LC [7,27,31,35]. Such composites are typically used for the fabrication of diffractive optical elements [7,23,29,31], lenses with tunable focal length [27,28,33], and waveguiding structures [32], as well as stabilization of blue phase LCs to room temperature to make them applicable in LCDs [30,36].

It was demonstrated that the coexistence of two different phases—nematic and isotropic—can occur when LC is heated to the phase transition temperature, as described by Landau-de Gennes’s theory [8]. In the case of LC materials confined in 1D space, the nematic–isotropic phase separation was observed to be periodic [43,44]. It was shown that the thermal stability of such self-assembled structures can be improved by adding nanoparticles, especially gold ones, to the LC host [43,45]. In our previous research, we demonstrated that the period of said structures correlates with the diameter of the confining space [43,45] and utilized a photopolymerization process to stabilize them to room temperature [45].

The research presented in this paper is focused on examining the possibility of manipulating the molecular arrangement of the polymer-stabilized self-assembled periodic LC structures by applying an external electric field. It is thought that such structures can operate as highly tunable fiber Bragg gratings [46] or long-period fiber gratings [47,48,49,50,51,52] and thus be utilized as sensors of electric and magnetic fields as well as temperature [50]. The propagation properties of the supposed fiber gratings will most likely depend on the spectrum of the light source—it is thought that the fiber Bragg grating behavior can be obtained for the near-IR range. In contrast, the long-period fiber grating behavior should be observable in visible light. Moreover, in the initial orientation of the LC, the structure possesses radial symmetry of molecular arrangement in the plane perpendicular to the propagation direction. Application of an electric field might introduce an easily detectable anisotropy in the structure, which should modify its propagation properties.

## 2. Experimental Details

The 1D periodic structures were fabricated by polymer stabilization of LC-based composite infiltrated into silica microcapillaries (20 μm internal diameter, and 125 μm external diameter). The material consisted of a nematic LC 5CB (4-cyano-4′-pentylbiphenyl, >99.5%, CAS 40817-08-1) in 98.9 wt% concentration doped with 0.1 wt% gold NPs (1–3 nm diameter) [3,19,53] and 1 wt% of a 95:5 mixture of monomer Bisphenol A dimethacrylate (>98%, CAS 3253-39-2, Sigma Aldrich, St. Louis, MO, USA) and photoinitiator 2,2-dimethoxy-2-phenylacetophenone (99%, CAS 24650-42-8, Sigma Aldrich) to allow for photopolymerization of the nematic–isotropic phase separation. The polymerized samples were stable at room temperature [45]. However, the molecular arrangement changed compared to the one obtained at the nematic–isotropic phase transition temperature. Our previous work provides a more detailed description of the preparation procedure and analysis of such polymer-stabilized periodic structures [45].

**Figure 1:**
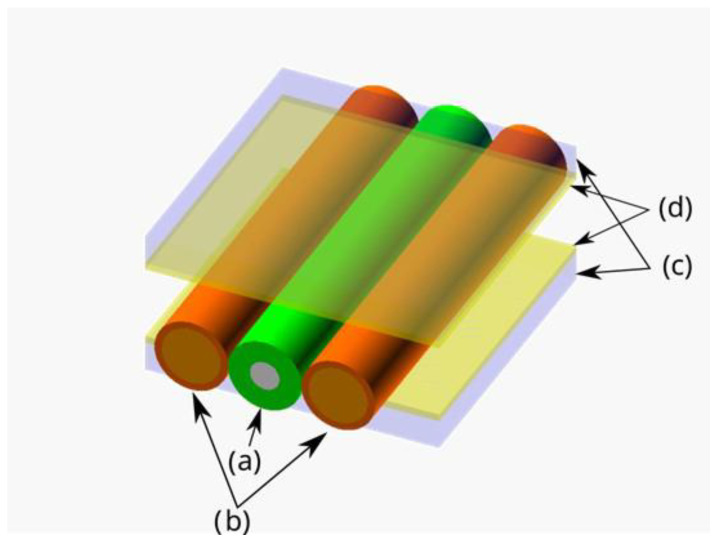
Scheme of the electrode setup: (a) the sample is positioned between two sets of electrodes: (b) metal wires (coated with an electric insulator) on the sides, and (c) glass plates with (d) ITO coating on top and bottom, facing towards the sample.

The polymer-stabilized samples were examined under the influence of an external electric field at 1 kHz frequency. All experiments were conducted at room temperature (around 20 °C). The setup consisted of two ITO-coated glass plates and two electrically isolated metal wires, with the external diameter and spacing between them slightly greater than the external diameter of the examined sample (~125 μm) so that it could be inserted into the setup. The wires acted both as additional electrodes and spacers between the glass plates. Such a setup, presented in Figure 1, allowed the induction of an electric field in two orthogonal directions with respect to the observation plane and thus enabled the reorientation of LC molecules in two different axes without the need for rotating the sample during analysis.

The observations of the sample were conducted under a polarizing microscope (Keyence VHX 5000, Poland, Warsaw, VHX-5000 ver 1.6.1.0, system ver 1.04) with the electrode setup positioned between crossed polarizers. The polarized optical microscopy technique allowed us to determine the LC’s molecular arrangement as the sample’s brightness, observed after the analyzer, changes according to the phase delay introduced by the LC. The electric field parallel to the microscope’s optical axis was induced by connecting the glass plates to the function generator. In the case of an electric field perpendicular to the optical axis, the wires were connected to the generator instead.

## 3. Results and Discussion

### 3.1. Results

For electro-optical analysis, the sample was inserted in the electrode setup presented in Figure 1, which was positioned under the polarizing microscope. The capillary was oriented in such a direction that planar nematic areas, with a director parallel to the long capillary axis, were also parallel to the transmitting axis of one of the polarizers. Such a configuration allowed for easy examination of changes in molecular arrangement.

**Figure 2:**
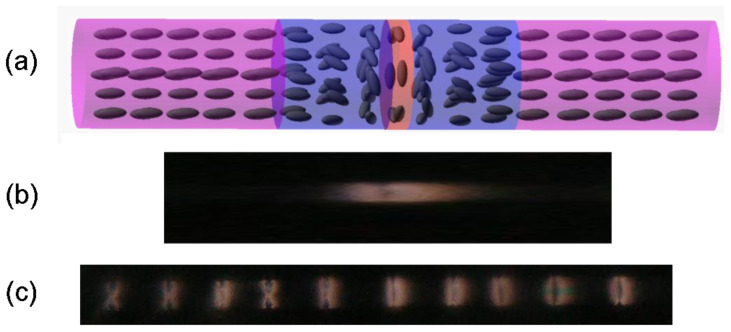
The top image (**a**) presents an initial molecular arrangement of the observed sample. The entire planar area is marked in violet, whereas the non-planar one is divided into two sections—blue and red—to simplify the analysis of LC reorientation. The middle image (**b**) is an enlarged and distorted element of a periodic structure, whose molecular arrangement is presented by the top image. The bottom image (**c**) presents the whole periodic structure in the initial state (without any electric field applied).

In the initial arrangement of the liquid crystal molecules, the non-planar areas appeared as a grain of coffee or an ‘X’ when no voltage was applied, which was consistent with the results presented in our previous paper [45]. To describe the molecular arrangement of LC inside the capillary under increasing external electric field strength as clearly as possible, the sample was divided into three types of sections, as shown in Figure 2a. The first type of section (Figure 2a red) is the center of the non-planar area, where LC molecules’ long axes (in a plane perpendicular to the long capillary axis) are arranged around the inner circumference of the capillary. The second type of section (Figure 2a, blue), also in a non-planar area, had LC molecules tilted, and their tilt angle changed from parallel to the long capillary axis (planar orientation) to perpendicular to the capillary long axis. The initially planar area, in which LC molecules’ long axes were parallel to the long capillary axis, is marked with violet in Figure 2a.

**Figure 3:**
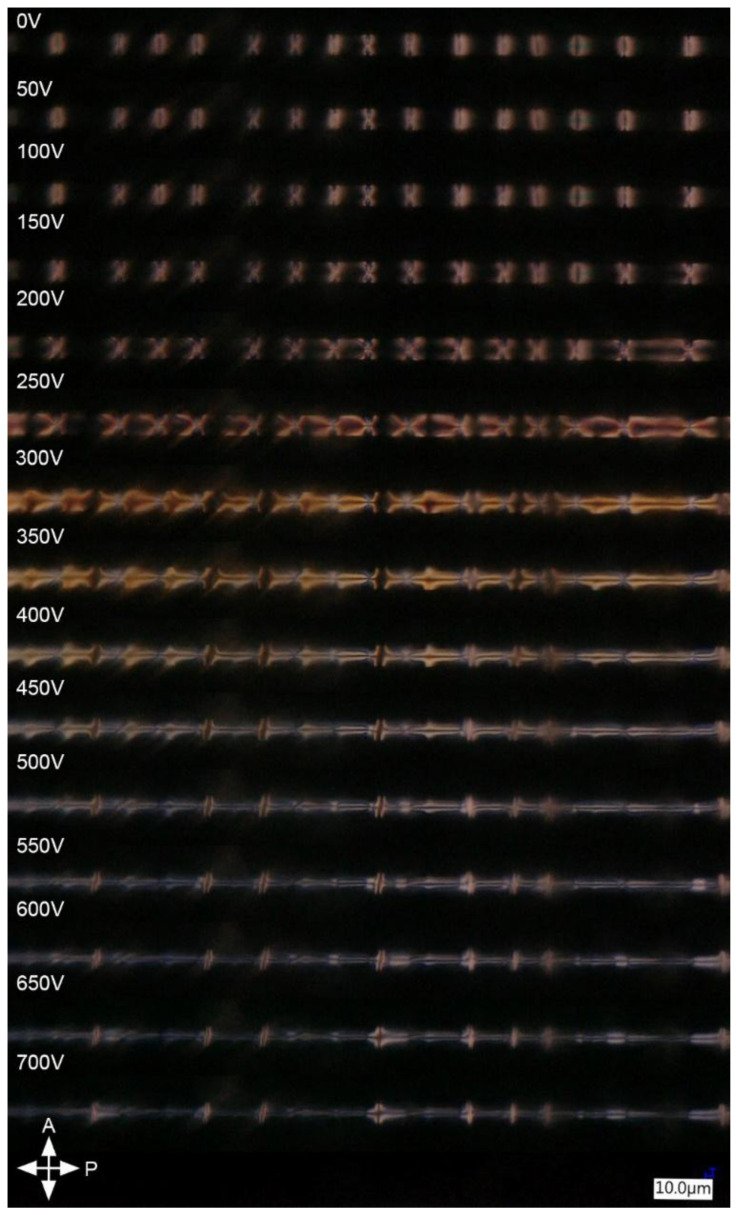
Reorientation of LC molecules in an external electric field, induced by high voltage AC generator in d-electrodes (Figure 1), parallel to the microscope’s optical axis. The distance between the electrodes is ~125 µm.

**Figure 4:**
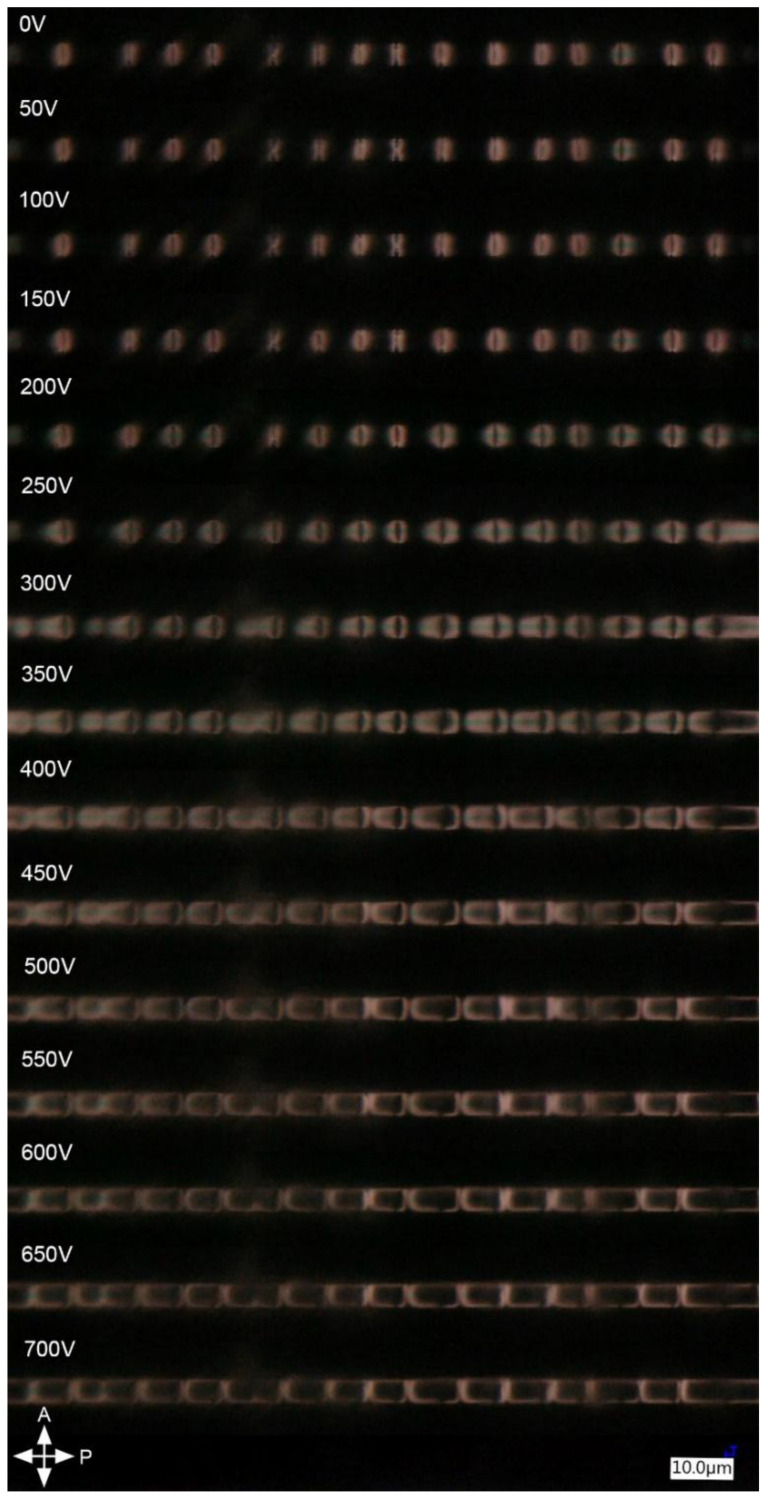
Reorientation of LC molecules in an external electric field induced by high voltage AC generator in b-electrodes (Figure 1), perpendicular to the microscope’s optical axis. The distance between the electrodes is ~125 µm.

When an external electric field was applied to the LC sample (Δε > 0), the molecules started to rotate to be parallel to the electric field vector. The reorientation of LC in the sample under two orthogonal directions of the electric field is shown in Figure 3 and Figure 4.

It was observed that the changes in the sample’s molecular arrangement in planar and non-planar areas began at different voltage values. Firstly, for lower amplitudes of AC voltage, reorientation occurred in the non-planar areas due to the pretilt of the LC molecules. On the other hand, the molecules in originally planar areas started reorienting after exceeding the threshold voltage (~2 V/μm, assuming 125 µm distance between electrodes), which was higher than typical for 5 CB due to additional anchoring resulting from the presence of the polymer network.

**Figure 5:**
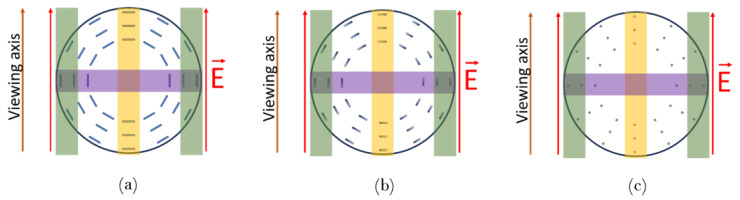
Cross-section of (**a**) the red section, (**b**) the blue section, and (**c**) the violet section of the sample (Figure 3) showing its initial molecular arrangement. The direction of the electric field $\vec{E}$, the optical axis of the microscope, and specific parts of the cross-section are marked to simplify the analysis of LC reorientation.

When analyzing the reorientation in the center of non-planar areas in Figure 3, one can see that no brightness changes were observed on the sides of the capillary (Figure 2a red) as the molecules there were already parallel to the direction of the electric field vector (Figure 5a, green region). A relatively similar arrangement, except for the center of the capillary, is present in the middle of the capillary, as shown in the violet region of Figure 5a. On the other hand, the molecules around these points started twisting toward the direction of the electric field for voltage values below the threshold, as the threshold voltage exists only for molecules perpendicular to the electric field. The lack of threshold was caused by the initial orientation of LC—the electric field was not perpendicular to the LC director except for the molecules marked with orange in Figure 5a. An interesting LC reorientation can be observed in the center of the red and blue section of Figure 2a—twists of LC molecules on the left and right side of the orange region (Figure 5a,b) had opposite directions, which caused a defect due to opposite bending of LC molecules around the orange region in Figure 5a,b or the on top and bottom of the capillary with respect to the viewing plane in Figure 3.

In the 0–200 V range, the described molecular rearrangement resulted in visible changes in the ‘coffee grain’ shape—it transitioned into an ‘X’ shape that was spreading towards the planar areas with increasing voltage. In the second part of the non-planar area (blue section in Figure 2a), the reorientation of LC molecules was complex and strongly dependent on the initial orientation of the molecules with respect to the direction of the electric field (Figure 5b).

At the edges of the capillary, marked in green in Figure 5b, LC molecules rotated around a constant axis, which was identical for all molecules in this region. In contrast, the molecules between the orange and green regions rotated in axes that were not parallel or perpendicular to the long capillary axis, and those rotation axes were not identical at every point of the capillary (they were not symmetric along the violet region). That is why parts of the sample marked with green in Figure 5b became dark for lower voltages than parts closer to the orange region. In the third type of section (Figure 2a violet), there were no changes in LC’s molecular arrangement below the threshold, and thus, in Figure 3, the planar areas were observed as black for voltages in the range 0–200 V. The LC reorientation in red and blue sections continued with increased voltage (250–700 V) until there was only a small defect in the middle of non-planar areas (an orange region in Figure 5b), which can be seen in Figure 3 as two bright horizontal stripes separated by a black stripe in the center. This defect was caused by the opposite directions of the LC twist in the right and left parts of the non-planar area. Moreover, the orientation changes were not the same across the non-planar areas when the electric field was applied due to interaction with the polymerized planar parts of the sample (violet section in Figure 2a).

### 3.2. Discussion

In violet sections (Figure 2a), where LC molecules were planarly arranged and polymerized, reorientation began after the voltage exceeded 250 V and did not occur identically across the whole section. Molecular reorientation started at the border with blue sections (Figure 2a), more specifically in the green regions in Figure 5c, and progressed towards the orange region. The molecules located further from the borders with blue sections began to reorient for higher voltages, and the reorientation continued until the center of the violet section was reoriented. The molecular arrangement in violet sections mimics exactly the arrangement in blue ones at lower voltages, including a defect in the orange regions that has been induced by the opposite twist direction. These are focused in the orange region due to the forced parallel orientation of the LC molecules to the electric field vector.

In the case of an electric field perpendicular to the setup’s optical axis (Figure 4), the molecular reorientation is, in principle, identical to the one shown in Figure 3. Change in the electric field vector corresponds to observing the sample from an orthogonal direction compared to Figure 3, which was technically simpler than changing the observation plane. For voltages in the range of 0–200 V, a minor change was observed in the non-planar areas—the bright areas expanded towards the planar ones. Moreover, the defects in molecular arrangement resulting in an ‘X’ shape (instead of ‘coffee grain’) disappeared due to reorientation in the center of the capillary. For higher voltages (250–400 V), the LC molecules at the borders with planar areas began to mimic the orientation of the non-planar ones, and the reorientation continued further into planar areas with increasing voltage. These changes in effective birefringence resulted in the observable expansion of the bright parts of the ‘coffee grain’, indicating rotation of the LC molecules toward the electric field vector.

After exceeding 350 V, this effect was followed by the expansion of the dark parts of the sample that were originally located in the red section and the extension of these into the blue section (Figure 2a), which means that in this area, the LC’s molecular arrangement is parallel to the electric field vector. For voltages above 500 V, the non-planar parts of the sample were observed to be bright only at the inner walls of the capillary. The changes in molecular arrangement of the planar areas in the 350–700 V range were first observed as a gradual change in brightness (Figure 2a violet) from dark to bright that started at the borders with the non-planar ones (Figure 2a blue). Next, these sections became dark again due to molecular reorientation originating from the fully reoriented red sections of non-planar parts of the sample (Figure 2a red). As a result, at 700 V, the sample became dark everywhere except for the inner walls of the capillary and the middle of the planar areas due to an insufficient voltage value for total reorientation. The same changes occur when the direction of the electric field is orthogonal to the viewing axis. To simplify, we can assume that the viewing axis changed, and through this, we observe changes in birefringence for different planes, so the same molecular reorientation has a drastically different effect on the observed image seen in Figure 3 and Figure 4.

**Figure 6:**
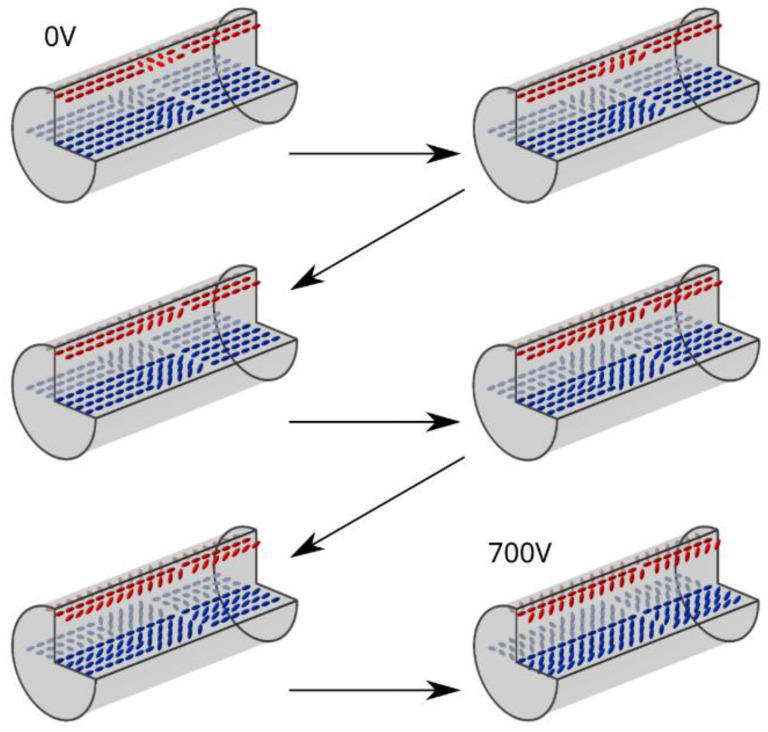
Reorientation of LC molecules in the electric field parallel to the microscope’s optical axis (Figure 3). The image in the top left corner shows the molecular arrangement with no electric field applied (0 V), and the one in the bottom right corner presents the molecular arrangement after reorientation for the highest voltage used during observations (700 V). The images in between demonstrate how the molecules reorient with increasing voltage until reaching 700 V.

**Figure 7:**
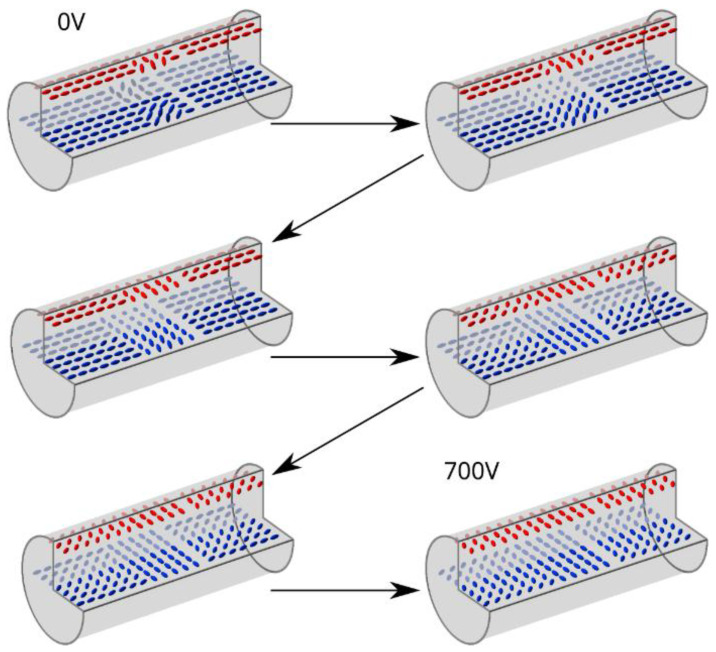
Reorientation of LC molecules in an electric field perpendicular to the microscope’s optical and long capillary axes (Figure 4). The image in the top left corner shows the molecular arrangement with no electric field applied (0 V), and the one in the bottom right corner presents the molecular arrangement after reorientation for the highest voltage used during observations (700 V). The images in between demonstrate how the molecules reorient with increasing voltage until reaching 700 V.

The entire reorientation process described above is schematically presented in Figure 6 and Figure 7. The 3D model focuses on the area around the coffee grain shown in Figure 2.

Despite drastic differences in observed images of the sample for two orthogonal directions of the electric field, the internal orientation of LC molecules is the same, excluding the 90° setup rotation. Figure 6 presents changes in a molecular arrangement under an electric field applied parallel to the optical axis. Similarly, Figure 7 presents changes in a molecular arrangement under an electric field perpendicular to the optical axis. In this case, the LC reorientation is easier to describe than it was in the previous case. The comparison of these two Figures highlights specific changes in LC orientation, especially changes seen in the middle of the capillary (for both vectors of the applied electric field). These results allowed us to determine characteristic features during reorientation, which can be observed for both directions of the applied electric field, and find minor changes that can be seen in one of the sample positions. With an electric field applied along the microscope’s optical axis, a characteristic change in LC orientation can be observed in the center of the capillary, which is caused by the opposite initial LC twist direction at the top and bottom of the capillary. However, most defects in the middle of the planar areas of the sample are not visible in this setup configuration. For specific electric field values applied perpendicularly to the microscope’s optical axis, the images show changes in LC orientation where no polarimetric change should be observed according to observations in the previous setup. It can be clearly seen that there are inconsistencies in observed images—in one setup, the image does not change from a certain value of applied voltage. In contrast, the same areas in the second setup still change with increased voltage. This implies that a defect in this type of area is focused near the capillary surface, specifically in the spot where this change cannot be easily seen, on the top and bottom of the yellow region of Figure 5c. As one can see, observations in only one electrode configuration do not provide a full insight into the reorientation of different parts of the periodic structure.

In addition to analyzing LC reorientation, the constancy of the structure’s period during reorientation was examined. For this purpose, a period detection algorithm was utilized, and it is the script made specifically to account for unusual shapes (grain of coffee, “x”, or parallel lines) observed under a polarizing microscope. The comparison of the period value before and during reorientation is shown in Table 1.

Despite drastic changes in molecular arrangement, the analyzed sample’s period remained constant due to the electrically induced formation of new periodic defects in LC orientation. The only changes that were present were in the sample’s effective refractive index. Such a result suggests that the proposed periodic structures have the potential to operate as highly tunable electric field sensors in the form of fiber Bragg gratings or long-period fiber gratings.

## 4. Conclusions

The presented results seem promising when it comes to utilizing the proposed polymer-stabilized periodic LC structures as electrically tunable fiber Bragg gratings or long-period fiber gratings. Reorientation of LC molecules under an external electric field is clearly visible within a wide range of voltage values, and the period value is preserved—only a change in the LC’s effective birefringence is observed. This results in modification of the effective refractive index of the fiber’s core, which should result in a change in the resonance wavelength. Further research within our group will focus on introducing light into such structures and characterizing their propagation properties under an external electric field and temperature.

## Figures and Tables

**Table 1:** Comparison of the structure period before and during reorientation for two orthogonal directions of electric field $\vec{E}$ with respect to the microscope’s optical axis.

	P [µm]	U(P) [µm]
$\mathrm{No}\;\vec{E}$ field	56	11
$\mathrm{Axial}\;\vec{E}$ field	54	12
$\mathrm{Perpendicular}\;\vec{E}$ field	53	10

## Data Availability

The original contributions presented in this study are included in the article. Further inquiries can be directed to the corresponding author.
